# Lack of tocilizumab effect on mortality in COVID19 patients

**DOI:** 10.1038/s41598-020-74328-x

**Published:** 2020-10-13

**Authors:** Gregory E. Holt, Mayank Batra, Mukunthan Murthi, Shweta Kambali, Kayo Santos, Maria Virginia Perez Bastidas, Huda Asif, Sara Haddadi, Sixto Arias, Mehdi Mirsaeidi

**Affiliations:** Division of Pulmonary and Critical Care, University of Miami, Miami, FL USA

**Keywords:** Diseases, Outcomes research

## Abstract

Off-label tocilizumab use in COVID-19 patients reflects concern for cytokine release syndrome. Comparison of matched COVID-19 pneumonia patients found elevated IL-6 levels correlated with mortality that did not change with tocilizumab administration. Correlating mortality with increased IL-6 doesn’t imply causality however lack of improvement by tocilizumab requires further clinical trial alterations.

## Introduction

The lack of proven medications for COVID-19 beget the widespread use of off label therapeutics based on theoretical efficacy. Early data correlated elevated levels of IL-6 with increased mortality^1^. This finding led clinicians to use the humanized anti-IL6 receptor antagonist, tocilizumab, to attenuate IL-6 levels in infected patients based on its proven benefit in patients suffering from cytokine release syndrome after chimeric antigen receptors (CAR)-T cell infusion^2,3^. In COVID-19, the question of whether elevated IL-6 levels reflect an overwhelming viral infection or directly cause immunopathology responsible for a patient’s poor outcome remains unanswered. We present our experience with tocilizumab in conjunction with the published literature to argue for the urgent need to understand a disease before carte blanche application of unproven therapies in single arm trials. We aimed to find the effect of tocilizumab on mortality of COVID19 in a well-matched population cohort study.

We performed a retrospective cohort study of matched patients admitted to the University of Miami Hospital with a confirmed diagnosis of COVID-19 approved by University of Miami.

Institutional Review Board (IRB #20,200,441). All procedures in this study were performed in accordance with relevant guidelines and regulations. The IRB waived a requirement for informed consent in the context of minimal risk research.

Tocilizumab was administered to patients after consultation with an Infectious Disease driven committee for COVID19 who required oxygen ≥ 4 L per minute via nasal cannula and had specific levels of at least 4 biomarkers; IL-6 > 40 pg/mL, CRP > 10 mg/dL, D- dimer > 1 mcg/mL FEU, ferritin > 1,000 ng/mL, or LDH > 350 units. Out of 250 confirmed COVID-19 patients, 32 (12.5%) received Tocilizumab during hospitalization and enrolled in the study. Patients received 400 mg tocilizumab as a single intravenous infusion. 24 patients received tocilizumab when started mechanically ventilation and rest on supplemental oxygen. Thirty patients who did not receive tocilizumab were matched for gender, age, ICU admission, and qSOFA score and included in the control group. Demographic, clinical, laboratory data, and in-hospital mortality data were collected from the medical records. For descriptive analysis of ordinal variables, we used Mantel–Haenszel methods. Continuous variables were reported as median and interquartile range (IQR). Medians of two groups were tested using Wilcoxon-Mann–Whitney test. We used Cox's proportional hazards model to analyze survival time and multivariate analysis to test the effect of each independent variable on mortality. A *p* value less than 0.05 was considered statistically significant.

**Figure 1:**
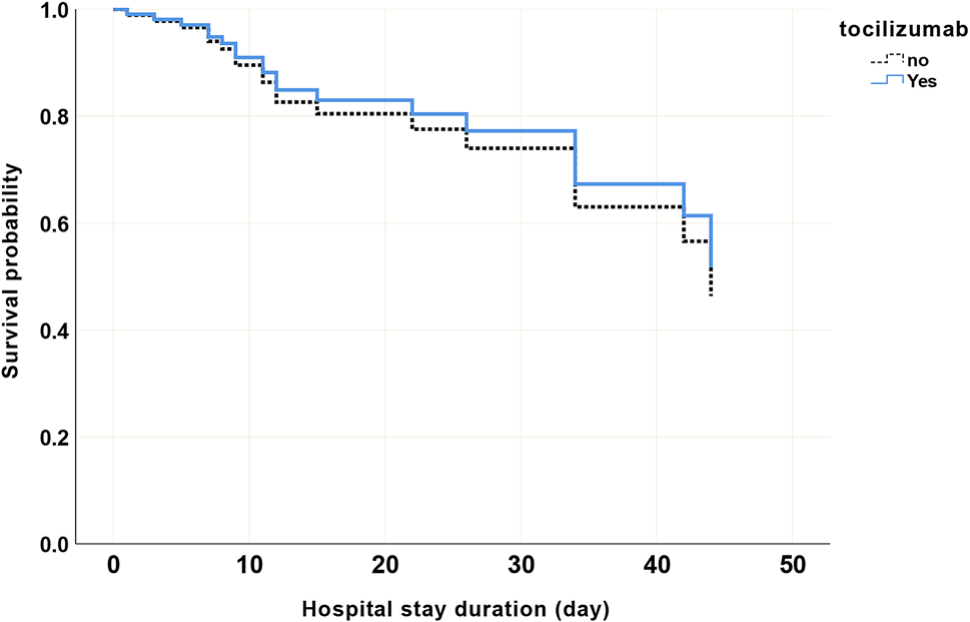
Cox regression showing no statistical difference in mortality between COVID 19 subjects treated and untreated tocilizumab therapy. Blue: shows treated with Tocilizuman, Black: shows untreated with Tocilizumab. Variables in the model: Age >  = 75, IL6 >  = 580, and Tocilizumab. *p* value, HR (95%CI) for Tocilizumab: 0.75, 0.9 (0.3–2.2).

**Figure 2:**
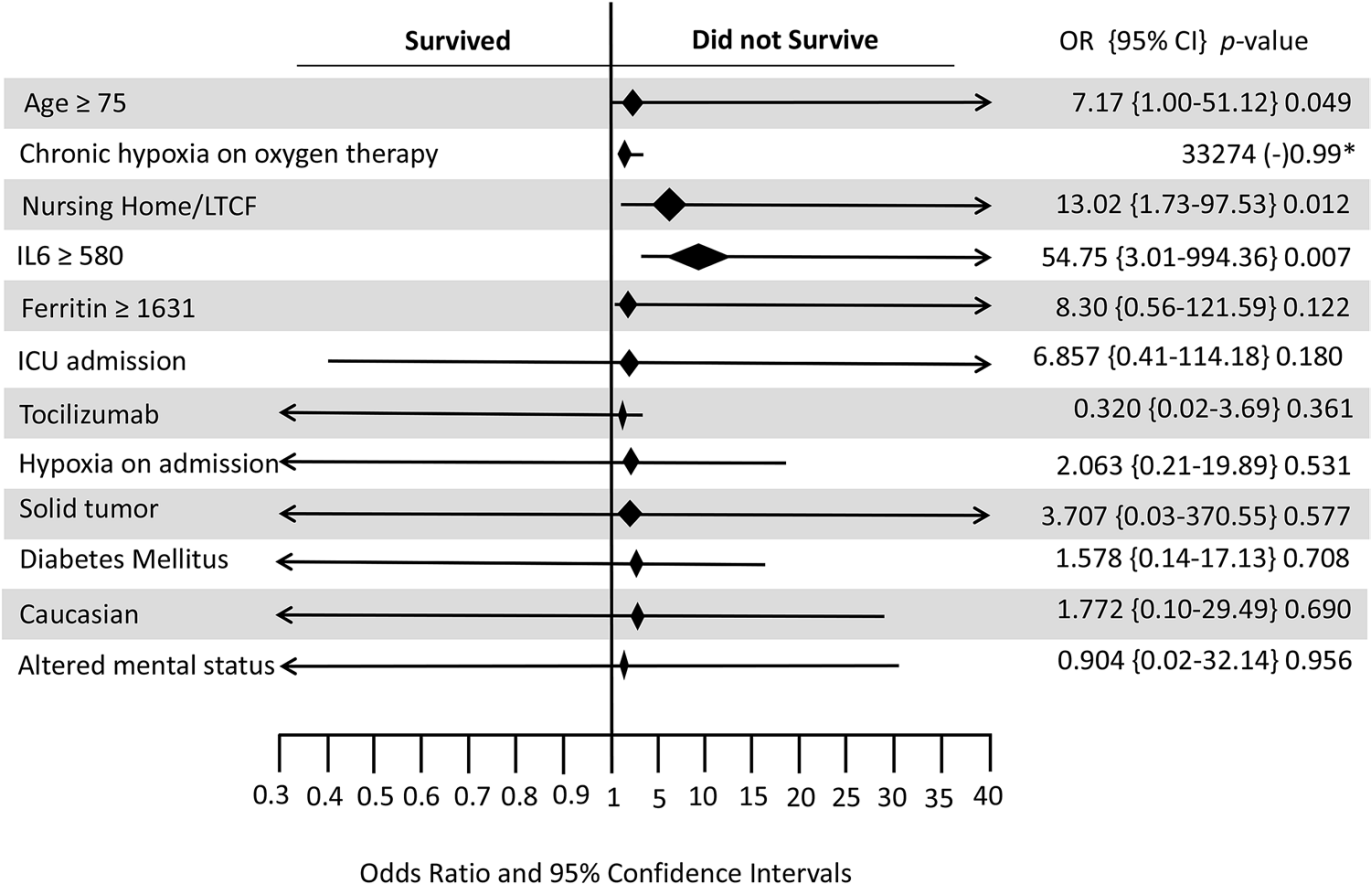
Forest plot showing the variables used in the multivariate model among COVID19 subjects. Hosmer lameshow score > 0.05, IL6 and Ferritin units: pg/ml.

Nineteen patients (30.6%) died during this study with 14 (22.6%) dying in the ICU. The majority of deaths occurred in subjects older than 75 years (OR 7.1, *p* = 0.049). Subjects with IL-6 levels over 580 pg/mL had an increased mortality (OR 54.7, *p* = 0.007) (Table 1). In multivariate analysis, tocilizumab administration had no discernible effect on mortality (OR 0.3, *p* value 0.36). Additional variables that were conclusive using the univariate analysis but did not hold up in multivariate analysis include; residents of nursing homes/long term care facility (OR 13.0, *p* = 0.12), diabetes mellitus (OR1.5, *p* = 0.70) solid tumors (OR 3.70, *p* = 0.57), ferritin ≥ 1631 (OR 8.3, *p* = 0.12), and altered mental status (OR 0.9, *p* = 0.95) (Fig. 1). Figure 2 shows survival analysis result and showing no significant difference in mortality between subjects treated and untreated with tocilizumab.

**Table 1:** Demographic and clinical characteristics, and outcomes of subjects with COVID19.

Demographics (N = 62)	Survived N (%)	Did not survive N (%)	*p* value
Age, Median (IQR) year	62 (42–82)	75 (58–92)	0.006
Age ≥ 75 (N = 15)	5 (11.6)	10 (52.6)	0.001
Male (N = 44)	30 (69.8)	14 (73.7)	0.754
Hispanics (N = 34)	23 (56.1)	11 (64.7)	0.545
White (N = 33)	20 (51.3)	13 (76.5)	0.085
Black (N = 15)	13 (33.3)	2 (11.8)	0.109
Asian (N = 2)	1 (2.6)	1 (5.9)	0.55
More than one race (N = 6)	5 (12.8)	1 (5.9)	0.452
Tobacco cigarette use (N = 39)	27 (62.8	12 (63.2)	0.978
Vaping (N = 51)	37 (86)	14 (73.7)	0.247
Alcohol (N = 50)	35 (81.4)	15 (78.9)	0.822
Marijuana (N = 49)	32 (74.4)	17 (89.5)	0.194
**Symptoms at or after 48 h of hospital admission**			
Temperature > = 100°F (N = 26)	18 (41.9)	8 (42.1)	0.986
Cough (N = 44)	32 (76.2)	12 (66.7)	0.447
Sore throat (N = 2)	1 (2.4)	1 (5.6)	0.553
Rhinorrhea (N = 4)	3 (7.3)	1 (6.3)	0.887
Dyspnea (N = 43)	30 (69.8)	13 (68.4)	0.916
Fever (N = 42)	29 (67.4)	13 (72.2)	0.713
Chills (N = 21)	13 (31)	8 (44.4)	0.318
Myalgias (N = 16)	10 (24.4)	6 (33.3)	0.478
Abdominal pain (N = 5)	4 (9.8)	1 (5.9)	0.636
Diarrhea (N = 5)	3 (7.3)	2 (11.1)	0.632
Nausea/vomiting (N = 7)	5 (12.2)	2 (11.8)	0.963
Altered mental status (N = 8)	3 (7.1	5 (29.4)	0.035
Pre-admission oxygen use (N = 5)	1 (2.4)	4 (21.1)	0.039
Inhaled steroid use (N = 6)	4 (9.5)	2 (11.1)	0.851
qSOFA, Median (IQR)	0.001 (0–0.5)	1 (0–2)	0.582
**Medications prior to hospital admission**			
Prednisone	6 (14.3)	2 (11.1)	0.741
ACE inhibitors	4 (9.5)	3 (17.6)	0.389
Angiotensin Receptor Blockers	7 (16.7)	3 (17.6)	0.928
Statins	10 (23.8)	7 (41.2)	0.187
Emergency Room visit within 12 months	10 (24.4)	4 (22.2)	0.857
Hospital admission within last 12 months	11 (26.8)	5 (27.8)	0.94
Nursing home/long term care facility residents	5 (11.9)	9 (47.4)	0.004
**Findings at Chest X-Ray or CT images obtained while in hospital**			
Ground glass opacities	9 (22.0)	5 (26.3)	0.71
Consolidations	10 (24.4)	8 (42.1)	0.168
Pleural effusions	5 (12.2)	5 (26.3)	0.181
Bilateral infiltrates	27 (65.9)	19 (31.7)	0.552
**Comorbidities**			
Chronic Obstructive Pulmonary Disease	4 (9.5)	1 (5.3)	0.58
Supportive oxygen before admission	16 (42.1	13 (68.4)	0.066
Congestive Heart Failure	1 (2.4)	2 (10.5)	0.211
Atrial fibrillation	4 (9.5)	2 (10.5)	0.903
Hypertension	23 (54.8)	14 (73.7)	0.167
Stroke	4 (9.5)	2 (10.5)	0.903
Dementia	2 (4.8)	1 (5.3)	0.933
Chronic Renal Failure	2 (4.8)	1 (5.3)	0.933
Diabetes Mellitus	10 (23.8)	13 (68.4)	0.02
Lymphoma	2 (4.8)	1 (5.3)	0.933
Solid tumor	1 (2.4)	4 (21.1)	0.039
**Laboratory findings at or within 48 h of admission to hospital**			
Procalcitonin ≥ 1.15 ng/mL	7 (16.3	5 (26.3)	0.36
Fibrinogen ≥ 649 mg/dL	2 (4.7)	1 (5.3)	0.918
IL-6 > 580 pg/mL	1 (2.3)	4 (21.1)	0.037
IL-6 Median (IQR) pg/mL	81.8 (0–264.9)	1197.3 (0–3738.9)	0.097
Ferritin > 1631 ng/mL	6 (14.0)	7 (36.8)	0.048
C-Reactive Protein ≥ 21	7 (16.3)	6 (31.6)	0.179
Mechanical ventilation use	21 (65.6)	15 (83.3)	0.19
Positive blood culture obtained after tocilizumab	5 (16.1)	2 (11.8)	0.683
Medications for COVID19			
Tocilizumab	22 (51.2)	10 (52.6)	0.915
Tocilizumab administration post-admission, Median (IQR) day	2 (0–5)	2 (0–6)	0.703
Tocilizumab for more than 4 days	6 (14.0)	2 (10.5)	0.711
Chloroquine/Hydroxychloroquine	31 (72.1	15 (32.6)	0.571
Macrolides	33 (76.7)	16 (84.2)	0.508
Steroids	26 (60.5)	10 (52.6)	0.565
**Outcome**			
Hospital stay duration, Median (IQR) day	22 (0–47)	11 (0–30)	0.102
ICU stay duration, Median (IQR) day	5 (0–24)	7 (0–21)	0.582
Patient delay*, Median (IQR) day	4 (0–10)	4 (0–10)	0.703
Physician delay**, mean (SD) day	3 (0–10)	4.0 (0–11)	0.609
Readmission	3 (7.0)	1 (5.3)	0.801
ICU admission	29 (67.4)	17 (89.5)	0.083

Our data show survivors had mean IL-6 levels of 177.9 + /- 227.9 whereas non-survivors had mean levels of 1384.2 + /- 1234.7 congruent with reports that elevated IL-6 levels are associated with poor outcomes in patients with COVID19 viral pneumonia^4,5^. The literature showing this correlation predominantly only measures admission IL-6 levels (most < 50 pg/mL) but does not follow them throughout the clinical course^5^. A recent study in Chest followed IL-6 levels after tocilizumab administration and found an immediate increase followed by a steady decrease but did not differentiate IL-6 levels between survivors and non-survivors^4^. Data from China found patients with rising IL-6 levels greater than 4000 pg/mL perished despite receiving tocilizumab^6^. These IL-6 levels begin to reach those seen in cytokine release syndrome (CRS) following CAR T cell infusion where levels reach a median value of 8,309 pg/mL for grade 4 or 5 CRS^7^.

The finding of an elevated cytokine level in disease does not prove causality regardless of the degree of correlation. However, rapid reversal of a clinical syndrome through use of cytokine specific blockade provides good data linking that cytokine to disease pathogenesis. Use of tocilizumab for CRS in patients treated with CAR T cells causes a dramatic improvement in disease usually within 48 h^8,9^. To date, tocilizumab has not had the same dramatic effect on reversing COVID-19 pneumonia like it does in CRS from CAR-T cell infusion despite papers suggesting possible benefit when compared to historical controls^10–14^.

Comparison of IL-6′s role in viral infections versus CRS after CAR-T cell infusion is informative. IL-6 is an important cytokine responsible for promoting anti-viral T cell responses, inhibiting viral replication and resolving inflammation to promote tissue repair^15^. However, mouse models of viral infections that artificially create supraphysiologic IL-6 levels (10,000 pg/mL) show viral persistence and increased immunopathology^16^. Immune stimulating agents in cancer, e.g. CAR T cells, are efficacious partly because they are impervious to negative immune regulation and allow unfettered immune reactions against tumor cells. Not surprisingly, the supraphysiologic IL-6 levels in CAR-T infusion both correlate with high tumor burdens^17^ and appear to be directly responsible for disease as administration of tocilizumab dramatically improves clinical status usually within 48 h^3^. Our data and another retrospective cohort trial of matched patients showed tocilizumab had no effect on mortality^18^.

There are several limitations to this retrospective cohort study including small sample size and retrospective cohort design. Tocilizumab was given based on clinical parameters and biomarkers *assumed* to indicate IL-6 mediated immunopathology. Although IL-6 is correlated with poorer outcomes, in COVID19 we do not know if, at what level or at what time point IL-6 leads to immunopathology. Tocilizumab may have failed to influence mortality because IL-6 may not be either responsible for, or the only cytokine involved in immunopathology. If IL-6 is responsible for CRS in COVID-19, it is unknown at what level or time point it changes from having anti-viral properties to causing immunopathology. Use of tocilizumab in patients regardless of IL-6 levels may have diluted out patients for whom tocilizumab may have benefited obscuring its effect on mortality. Tocilizumab would not help patients who did not produce pathologic IL-6 levels but could be detrimental if lower IL-6 levels were necessary to fight the viral infection^15^.

We believe the need to “do something” has superseded the need to evaluate disease to apply clinical trials based on data. We argue that evaluation of immune parameters in COVID19 patients need to first be studied to ensure that IL-6 is involved in immunopathology and second to determine at what level or time point in the clinical course of infection, IL-6 produces immunopathology. Serial measurements of key cytokines in COVID19 may characterize IL-6 and additional cytokine levels to correlate with clinical outcomes, before administration of tocilizumab. After measuring the levels of cytokines in the clinical course of a COVID19 infection, trials should be attempted to apply immune modulating therapy to patients for whom the immune system is causing disease via dysregulation.
